# Data on meq gene sequence analysis of Ludhiana MDV isolates

**DOI:** 10.1016/j.dib.2016.08.052

**Published:** 2016-08-31

**Authors:** Mridula Gupta, Dipak Deka, Ramneek

**Affiliations:** a Animal Biotechnology, School of Animal Biotechnology, GADVASU, Ludhiana 141004, India; b School of Animal Biotechnology, GADVASU, Ludhiana 141004, India

**Keywords:** Phylogenetic analysis, Sequence analysis, Meq gene

## Abstract

The data described are related to the article entitled “**Sequence Analysis of Meq oncogene among Indian isolates of Marek׳s Disease Herpesvirus**” M. Gupta, D. Deka, Ramneek, 2016. Seven meq genes of Ludhiana Marek׳s disease virus (MDV) field isolates were PCR amplified by using proof reading Platinum Pfx DNA polymerase enzyme, sequenced and then analyzed for the distinct polymorphisms and point mutations. The sequences were named as LDH 1758, LDH 2003, LDH 2483, LDH 2614, LDH 2700, LDH 2929 and LDH 3262. At this point, their deduced Meq amino acid sequences were compared with GenBank available already sequenced meq genes worldwide in their deduced amino acid form to study their identity/similarity with each other.

**Specifications Table**

**Table:** 

Subject area	*Biology*
More specific subject area	*Molecular Biology, Bioinformatics*
Type of data	*Figure, Table*
How data was acquired	*PCR amplification, Cloning and Sequencing of Meq gene of 7 MDV local isolates*
Data format	*Raw, analyzed*
Experimental factors	*Full deduced Meq amino acid sequences were aligned in DNASTAR LasergeneMegAlign Software and analyzed for the construction of phylogenetic tree in MEGA5.*
Experimental features	*Analysis was done with BLAST, MegAlign, Clustal W, MEGA5.20*
Data source location	*Ludhiana, Punjab, India*
Data accessibility	*Data are available in this article and at GenBank via accession numbers: GenBank:*KF895029.1*for LDH 1758, GenBank:*KF895030.1*for LDH 2003,GenBank:*KF895031.1*for LDH 2483, GenBank:*KF895032.1*for LDH 2614, GenBank:*KF895033.1*for LDH 2700, GenBank:*KF895034.1*for LDH 2929, GenBank:*KF895035.1*for LDH 3262*

**Value of the data**

• The data provide full length meq gene sequences of seven Marek׳s disease virus (MDV) field isolates which may be useful in comparative analysis of nucleotide, amino acid sequence similarities and phylogenetic studies with MDV strains available worldwide.
• The protein primary structures can be further used to obtain three-dimensional models and antigenic index of Meq proteins, useful to compare the domain organization of the deduced Meq amino acid of MDV strains worldwide.
• The presence of different number of proline (PPPP) repeats in the Meq protein may be correlated with oncogenicity as well as virulence of the MDV isolates.

## 1 Data

**Fig. 1:**
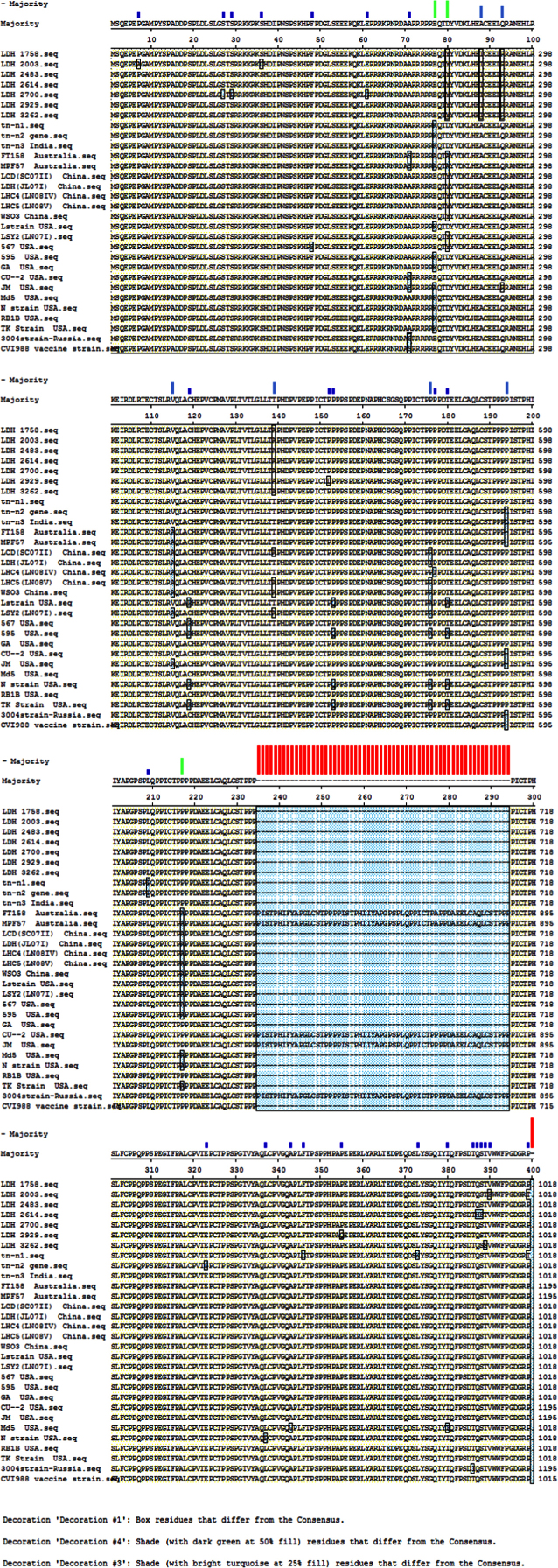
Alignment view using Meg Align DNASTAR Lasergene Software for Meq sequences i.e. LDH 1758, LDH 2003, LDH 2483, LDH 2614, LDH 2700, LDH 2929, LDH 3262, tn-n1, tn-n2, tn-n3, FT158, MPF57, LCD (SC07II), LDH (JL07I), LHC4 (LN08IV), LHC5 (LN08V), WSO3,LSY2 (LN071), L strain, 567 strain, 595 strain, GA strain, CU-2 strain, JM strain, Md5 Strain, N strain, RB1B, TK strain, 3004 strain and CVI 988.

The data include deduced amino acid Meq sequences of MDV isolates, sequence alignments and comparative analysis (Fig. 1). The alignment of all Meq amino acid sequences of all Ludhiana isolates i.e. LDH 1758, LDH 2003, LDH 2483, LDH 2614, LDH 2700, LDH 2929 and LDH 3262 with different Meq amino acid sequences worldwide is shown in Fig. 1. This data revealed that seven Meq amino acid sequences of Ludhiana isolates had the highest sequence similarity with LSY (LN07I), LCD (SC07II), CVI 988/Rispens vaccine strain and mCU-2 strain. The values of similarity between the sequences are reported in [1].

## 2 Experimental design, materials and methods

The PCR amplification of *Meqgene* sequence was carried out using specific primers and proof reading Platinum PfxDNA polymerase from seven Marek׳s Disease Virus (MDV) Ludhiana isolates. Cloning was done in suitable vector (pJET /1.2 blunt vectors)as mentioned in the research article. In order to obtain the full length of *Meq* gene sequences, selected recombinant clones were sent for sequencing by commercial outsourcing with sequencing primers as given in Table 1. The full length *Meq*gene sequences were analyzed by using BLASTn (Basic Local Alignment Search Tool) program [2]. These sequences were further analyzed by using DNASTAR Lasergene v 10 software [3].

The *Meq* gene sequences from the GenBank available different MDV strainswere used for alignments to compare the sequences with LDH 1758, LDH 2003, LDH 2483, LDH 2614, LDH 2700, LDH 2929 and LDH 3262 isolates as shown in Table 2. These sequences were aligned by using Clustal W method of Meg Align program as shown in Fig. 1. The m*eq* gene sequences of the seven Ludhiana isolates were submitted to the GenBank and the accession numbers obtained were GenBank: KF895029.1 for LDH 1758, GenBank: KF895030.1 for LDH 2003, GenBank: KF895031.1 for LDH 2483, GenBank: KF895032.1 for LDH 2614, GenBank: KF895033.1 for LDH 2700, GenBank: KF895034.1 for LDH 2929 and GenBank: KF895035.1 for LDH 3262.

## Figures and Tables

**Table 1:** Sequencing primers of pJET1.2 vector.

Primer binding sites		
pJET1.2 forward sequencing primer	Sequencing of insert, colony PCR. Sequence: 5′- CGACTCACTATAGGGAGAGCGGC- 3′	310-332
pJET1.2reverse sequencing primer	Sequencing of insert, colony PCR. Sequence: 5′- AAGAACATCGATTTTCCATGGCAG- 3′	428-405

**Table 2:** Meq sequences of MDV strains obtained from GenBank for comparison with sequences of Ludhiana isolates.

MDV strain/Sample	Origin	Accession number
tn-n1	India (South)	HM749324.1
tn-n2	India (South)	HM749325.1
tn-n3	India (South)	HM749326.1
FT158	Australia	EF523771.1
MPF57	Australia	EF523774.1
LCD (SC07II)	China	HQ658611.1
LDH (JL07I)	China	HQ658614.1
LHC4 (LN08IV)	China	HQ658618.1
LHC5 (LN08V)	China	HQ658619.1
WSO3	China	HQ638152.1
LSY2 (LN071)	China	HQ658625.1
L strain	USA	AY362717.1
567 strain	USA	AY362709.1
595 strain	USA	AY362715.1
GA strain	USA	M89471.1
CU-2 strain	USA	AY362708.1
JM strain	USA	HM488348.1
Md5 Strain	USA	AF243438.1
N strain	USA	AY362718.1
RB1B	USA	AY243332.1
TK strain	USA	AY362721.1
3004 strain	USA	EU032468.1
CVI988	Commercial vaccine	AF493555.1
