# Supplementary material for: Data on meq gene sequence analysis of Ludhiana MDV isolates
Source: Data Brief. 2016 Aug 31;9:231–5. doi: 10.1016/j.dib.2016.08.052 (PMC5021795; doi:10.1016/j.dib.2016.08.052)
Supplement: Supplementary file 1 — Supplementary material [file mmc1.doc]

Conflict of Interest: Nil

Manuscript Title : **Data on meq gene sequence analysis of Ludhiana MDV isolates.**

Authors’ name

Mridula Gupta

Dipak Deka

Ramneek
